# Floral Roles in Hummingbirds‐Mediated Indirect Plant Interactions in Tropical Andean Communities

**DOI:** 10.1002/ece3.72200

**Published:** 2025-09-30

**Authors:** Ann Frías‐Romero, Boris A. Tinoco, Bryan G. Rojas, Ariana Vélez, Samara Zeas‐Bermeo, Catherine H. Graham

**Affiliations:** ^1^ Programa de Posgrado en Recursos Naturales Renovables Universidad del Azuay Ecuador; ^2^ Escuela de Biología Universidad del Azuay Ecuador; ^3^ Swiss Federal Institute for Forest Snow and Landscape Research WSL Birmensdorf Switzerland

**Keywords:** floral traits, hummingbird‐mediated networks, plant–plant interactions, pollen grains, pollination networks, tropical Andes

## Abstract

In pollination networks, indirect plant–plant interactions mediated by their shared pollinators can shape community dynamics and species fitness; yet, the influence of floral traits on species roles remains unclear, particularly in diverse ecosystems like the tropical Andes. We studied hummingbird‐mediated interactions among 31 flowering plants in three high‐elevation shrubby habitats located in southern Ecuador. During August and November 2022, and January 2023, we collected stigma samples and constructed weighted interaction networks linked by heterospecific pollen grains. Species roles were determined by defining if they were donors or receivers of pollen, measured by node degree out and node degree in, respectively. We also explored the association between the abundance of flowers and different floral traits and species roles. Finally, we assessed the potential influence of floral roles on species fitness by calculating the ratio of conspecific‐to‐heterospecific pollen grains observed in each species. We found that the identity of donor and receiver species was highly dynamic across habitats and time. Receiver species were characterized by being highly abundant, while donor species presented high levels of nectar production. Receiver species received more heterospecific pollen than conspecific pollen, indicating that the pollen‐sharing roles of species could have fitness consequences. Our findings highlight the importance of some floral traits and abundance in shaping floral roles and their potential fitness consequences in hummingbird‐mediated indirect interactions.

## Graphical abstract

Hummingbirds connect plants by moving pollen. Some flowers primarily donate, while, others primarily receive, and these shifting roles influence plant reproductive success depending on abundance and nectar rewards, shaping plant reproduction in the tropical Andes. Chord diagrams represent heterospecific pollen transfer among 31 flowering species across sites: La Tranca (A, B & C), Aguarongo (D, E & F) and El Gullan (G, H & I), and seasons: August 2022 (A, D & G), November (B, E & H) and January (C, F & I). Each node represent a floral species. The size of the nodes and arrows denote the prevalence of interspecific pollen transfer. The direction of the tops of the arrows indicate the direction of the interaction.

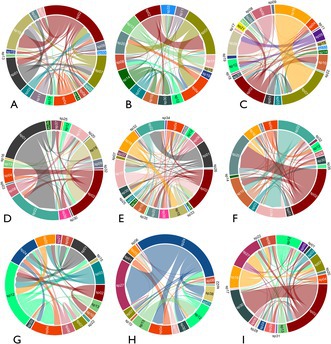

## 1 Introduction

Indirect interactions among species are known to have important fitness consequences (Strauss 1991; Werner and Peacor 2003), yet they remain less explored in comparison to direct interactions. In pollination, indirect interactions occur between co‐occurring plants that share the same pollinators (Carvalheiro et al. 2014). These interactions are mediated by plants competing to attract pollinators and the consequences of heterospecific pollen deposited by shared pollinators (Arceo‐Gómez 2021). While recently there have been major advances in understanding the structure of indirect interactions using network analysis tools (Bergamo et al. 2021; Carvalheiro et al. 2014; Fang and Huang 2013), the influence of flower traits on plant species' indirect interactions is still unclear, especially in species‐rich systems such as the tropical Andes.

Flowering plants can have different floral roles in indirect interaction networks and function as overall receivers or donors of pollen within communities. Receiver species obtain high amounts of heterospecific pollen from other species in the community, while donor species deliver their pollen to other species of the community (Lanuza et al. 2021). Indirect interaction networks often exhibit highly asymmetric patterns (Vázquez et al. 2007), where only a few species act as overall receivers or donors of pollen (Fang and Huang 2013; Peuker et al. 2020). Such asymmetry fosters cohesion and resilience within the network, promoting the persistence of rare species and minimizing competition among species (Bascompte et al. 2003; Bastolla et al. 2009). However, these asymmetric patterns also suggest that pollination interactions may be structured by a select group of plants that can have an important role in plant community dynamics and stability (Suárez‐Mariño et al. 2019).

The pollen sharing roles of species can be influenced by the abundance of flowers and the functional traits of the interacting species. For instance, the abundance of flowers can determine the amount of pollen potentially available in the community (Wei et al. 2021) and thus may influence the role of the species in the community. While highly abundant flowers have a high frequency of interactions (Vázquez et al. 2007) and are thus likely to donate pollen to the community (Arceo‐Gómez 2021; Fang and Huang 2013), rare species benefit from being in dense conspecific patches and receiving pollen through interspecific facilitation with abundant species (Bergamo et al. 2020). Moreover, floral traits can filter the pollinator visiting a plant species and determine the interactions among co‐flowering plants (Bergamo et al. 2019; Lunau et al. 2011). For instance, floral tube length and corolla width can influence the type of pollinators visiting a flower (E‐Vojtkó et al. 2020) and the amount of heterospecific pollen shared among species (Muchhala and Thomson 2012). Flowers with short floral tubes and wide corolla flowers often have a receiver role because they receive visits from multiple pollinators (Muchhala 2007; Stang et al. 2006). Conversely, long‐tubed flowers and narrow corollas attract mostly specialized pollinators (Aigner 2004; Dalsgaard et al. 2021; Sonne et al. 2020), reducing the diversity of pollinator visits (Benadi and Pauw 2018); as a result, they may receive less pollen and are likely to donate more pollen to the community. Another potentially important floral trait is the exertion of stamens and stigmas. Stigmas represent the receptive tip of flowers where pollen arrives, while anthers contain pollen grains to be shared with pollinators. Increased exposure of stamens may result in more pollen donation (Li et al. 2022), whereas greater stigma exposure can lead to higher pollen reception. Finally, the sugar concentration and the volume of nectar in a flower can determine pollinator visitation rates and influence pollen reception or donation (Heiling et al. 2021; Kim et al. 2011). Species with highly rewarding nectar for pollinators may have high visitation rates and have donor roles in the community, while those with less attractive nectar may act as receivers due to low visitation rates.

The roles of species within networks can have fitness consequences, particularly in plant–plant interactions, where the ultimate outcome depends on the number of conspecific pollen deposited on the stigmas that are able to fertilize ovules (Arceo‐Gómez 2021; Lopes et al. 2022). As a result of obtaining pollen grains, receiver species may accumulate heterospecific pollen, which can have negative fitness costs (Streher et al. 2020). Heterospecific pollen can prevent the germination of conspecific pollen and reduce reproductive success (Morales and Traveset 2008; Moreira‐Hernández and Muchhala 2019). For donor species, widely distributing pollen increases the chances of their pollen reaching another individual of the same species (Tur et al. 2016). Nonetheless, being a donor species can also entail fitness costs related to pollen waste (Muchhala et al. 2010; Muchhala and Thomson 2012).

In the tropical Andes, hummingbirds are highly diverse and specialized nectar‐feeding birds, playing a crucial role in pollinating a significant number of plant species (Barreto et al. 2024; Stiles 1981). Thus, the plant‐hummingbird system has been used as a model to investigate the evolutionary, historical, and ecological factors that shape the assembly of species within networks of interacting species (Crespo et al. 2022; Graham et al. 2012; Sonne et al. 2020; Tinoco et al. 2017). We studied the indirect interactions among flowering plants that share hummingbird pollinators in high elevation shrubby habitats located in the southern Ecuadorian Andes. We built indirect interaction networks in order to (1) identify the floral roles (receivers or donors) of the species, (2) evaluate the importance of floral abundance and floral traits in these roles, and (3) explore the potential fitness consequences for plant species with different roles. We collected 1454 samples of gynoecium from among 31 flower species, from which we expected to find (1) some species playing a defined floral role, but not the majority, (2) low‐abundance flowers functioning as receivers, and high‐abundance flowers as donors, (3) donor species with floral traits related specifically to hummingbird pollination (i.e., long corollas and narrow corollas), and receiver species with floral traits that allow pollen deposition from a variety of groups of pollinators, (4) negative fitness consequences for receiver species due to the accumulation of heterospecific pollen grains.

## 2 Methodology

### 2.1 Study Area

**FIGURE 1:**
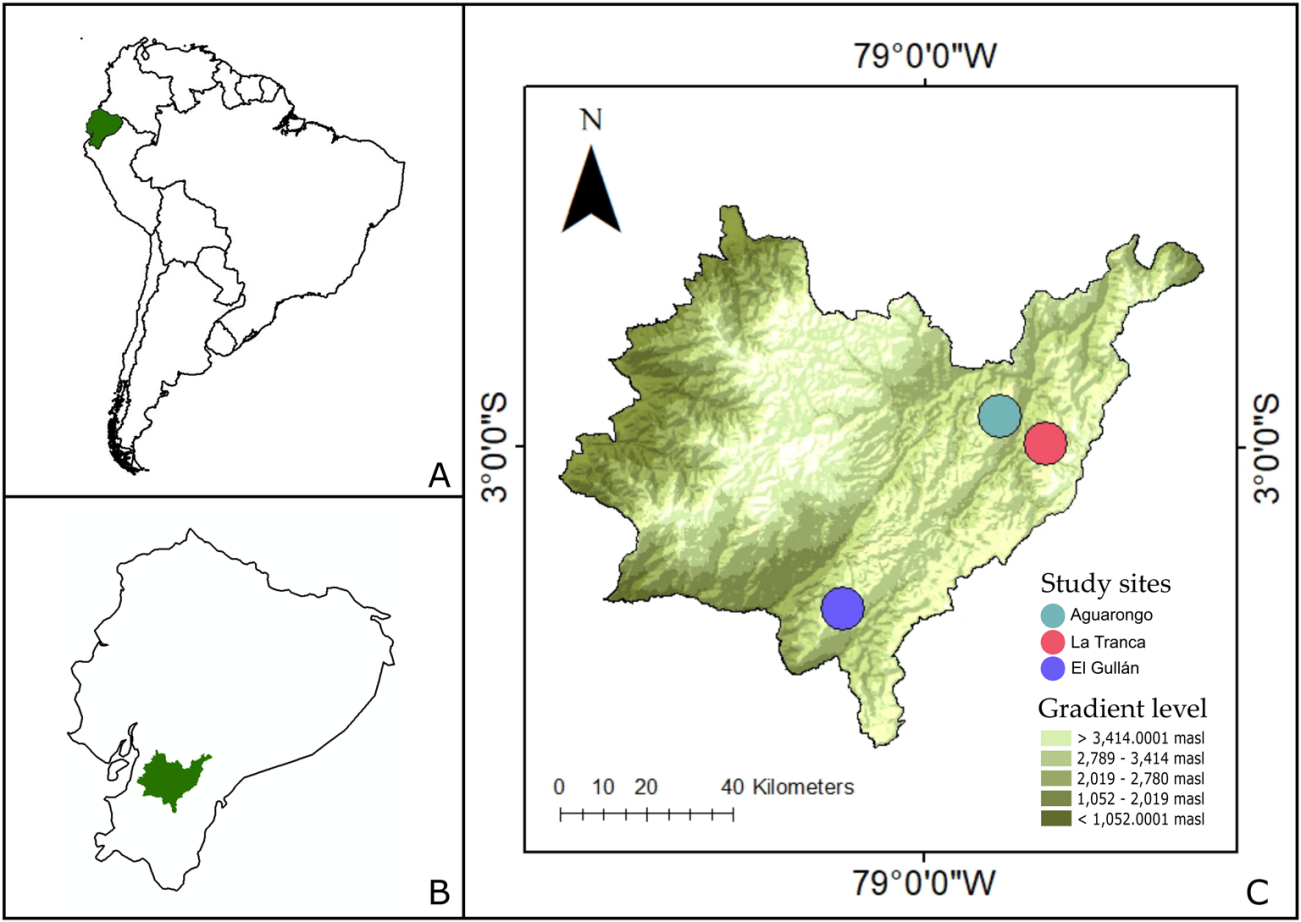
Location of the study area and sampling sites in southern Ecuador. Panel (A) shows the location of Ecuador within South America, panel (B) highlights the Azuay province within Ecuador, panel (C) shows a digital elevation model of Azuay province showing the altitudinal gradient and the location of the three study sites: El Gullán, Aguarongo and La Tranca. Elevation ranges are represented in different shades of green, from lowlands (light green) to highlands (dark green). A scale bar and north arrow are included for spatial reference.

Fieldwork was conducted in three sites located between 2900 and 3100 m.a.s.l. in the Southern Andes of Ecuador: El Gullán (3000 m asl), Aguarongo (3160 m asl), and La Tranca (3060 m asl) (Figure 1), located at least 10 km apart. Previous studies in the tropical Andes detect significant floristic turnover over similar spatial scales, driven by elevation, edaphic variation, and landscape heterogeneity (Cuesta et al. 2017), supporting the expectation that distinct floral communities occur at each site, justifying their treatment as ecologically independent units. The average temperature in the region ranges from 14°C to 20°C (MAGAP 2013), while average rainfall varies across sites: El Gullán from 400 to 600 mm per year, Aguarongo from 700 to 800 mm per year, and La Tranca from 1200 to 1500 mm per year (MAGAP 2012). The region experiences a bimodal rainfall pattern, characterized by two periods of substantial precipitation, from March to April and October to December, and one season with comparatively lower rainfall from June to August (Celleri et al. 2007). Fieldwork was carried out in three sampling periods in each site: August, November 2022, and January 2023.

All sites are dominated by native shrubs, including species that are known to be important nectar resources for hummingbirds such as *Oreocallis grandiflora, Barnadesia arborea, Fuchsia* sp., *Salvia* sp., *Macleania rupestris, Brachyotum confertum, Tillandsia* sp., or *Viola arguta* (Crespo et al. 2022). The sites chosen were mostly undisturbed by human activities; however, in Aguarongo and La Tranca, there are a few cattle trails that crossed our sampling transects.

### 2.2 Study Design and Sampling

This study was based on interactions among flowering plants measured by the presence and abundance of heterospecific pollen found on flower stigmas of co‐flowering plant species visited by hummingbirds. We focused on species known to interact with hummingbirds in the montane forests of Azuay, drawing on local studies in pollination ecology (Crespo et al. 2022; Knowlton et al. 2022; Tinoco et al. 2017; Vélez et al. 2025). Although these species may also be visited by other pollinators, most exhibit traits associated with the ornithophilous pollination syndrome, suggesting that hummingbirds are their primary pollinators. Consequently, we assume that indirect interactions among these plants are mainly mediated by hummingbirds. This assumption is supported by previous findings indicating that species with ornithophilous traits predominantly interact with hummingbirds, and indirect interactions among them are likely mediated by these interactions (Bergamo et al. 2017). The identification of species was helped by the use of a local field guide for plants (Arias et al. 2022). At each study site, we placed three 100 × 10 m transects, located at least 100 m apart, where we counted the flowers of each individual of all flowering plant species found in the transect. This information was used later to determine the number of stigmas to be sampled per flowering species by assigning each species to an abundance tercile based on its overall abundance at each sampling period and site. The least abundant species occupied tercile one and the most abundant occupied tercile three. Our samples were the stigma of the flowers, and we took three samples per individual according to abundance terciles. For tercile one, species with low abundance, we took samples from 2 individuals (6 samples); for tercile two, species with medium abundance, we took samples from 3 individuals (9 samples); and for tercile three, we took samples from 5 individuals (15 samples).

Stigmas were collected directly in the field and placed on slides coated with fuchsin dye, which were later examined under a microscope for pollen grain identification. Each slide was observed under 40× magnification, with the cover slip (measuring 25 × 25 mm) serving as the area to be examined for pollen. The field of view of the microscope was swept across the entire cover slip from left to right, covering each millimeter of the slip. To facilitate pollen grain identification, we compiled a library consisting of plant species present at our study sites. This library incorporated information from previous work in the study area (Jaramillo Espinosa 2019; Nieto Orellana and Silva Alemán 2012), complemented by our work.

### 2.3 Network Construction and Species' Roles

We constructed nine indirect interaction networks, three networks per site per sampling period. The network nodes represent species, genera, or families, depending on the level of identification we were able to reach, and the arrows connecting nodes (plant taxa) demonstrate the direction and prevalence of interspecific pollen transfer as determined from heterospecific pollen grains encountered on the stigmas of each plant species (Function *graph_from_adjacency_matrix* in R (Csárdi et al. 2024)). The networks included up to 38 nodes: 27 identified at the species level, 8 at the genus level, and 3 at the family level. To weight networks, we used the median value for each distinct heterospecific pollen type encountered in the stigmas of each plant species. The median value was used because it allowed us to capture a central tendency in the asymmetric distribution of pollen grain counts. Using the constructed networks, we created chord diagrams (Function Chord diagram in R (Gu et al. 2014)) to enhance the visualization of indirect interactions, and calculated node degree—in and out—using the function *degree()* from the igraph package (Csárdi et al. 2024).

To determine the pollen sharing roles of the different species, we calculated the weighted node degree in and weighted node degree out, considering the number of links entering and leaving a node, respectively (Foster et al. 2010; Leicht and Newman 2008). Species with higher values of weighted node degree‐in than others in the community can be identified as receiver species, while species with higher weighted node degree‐out compared to others can be considered donor species (Fang and Huang 2013).

### 2.4 Floral Traits

For the sampled species (Table S1) we gathered information on floral traits that are known to influence patterns of pollen sharing. This data was taken from a local database with at least 10 individual measurements for each species (Fernández and Frías Romero 2021). Corolla opening can function as a morphological barrier to pollinators (Muchhala 2007), and was measured at the widest part of the corollas, where the flower restricts access to pollinators. Floral tube length is a morphological barrier to pollinators (Sonne et al. 2020), and was measured as the distance from the base of the flower to the tip of the corolla. Exertion of stamen is the difference between stamen length and floral tube length. Exertion of stigma is the difference between stigma length and floral tube length. In these last traits, positive values represent stamen or stigmas extending beyond the floral tube, whereas negative values indicate that the stamen or stigma is situated inside the floral tube. Finally, nectar production and nectar concentration represent the reward offered to pollinators (Hainsworth and Wolf 1972; Kingsolver and Daniel 1983). Nectar production was measured using microcapillary tubes by recording the length of nectar drawn into the tube. Nectar concentration was determined with a handheld refractometer (%w/w). To obtain standardized measurements, flowers were first measured ‐both nectar production and concentration‐ < and then enclosed with fine mesh (tulle) to exclude pollinators. After 24 h, nectar volume and concentration were measured. Another important floral trait that could influence pollen sharing is the amount of pollen produced by each species and how the pollen is placed on the pollinator's body (Stewart and Dudash 2016). However, we were not able to obtain data on these traits due to the amount of work required to count pollen grains and assess placement patterns.

As indicated in the section Network construction and species' roles, some species were not identified at the species level using pollen samples. For those species, we used mean trait data of the genus. That was the case for eight species (*Fuchsia* spp., *Rubus* spp., *Tillandsia* spp., *Centropogon* spp., *Castilleja* spp., *Brugmansia* spp., *Berberis* spp., *Nasa* spp.). We are aware that there could be large variation in floral traits within species of the same genus, but we minimized that variation by using information of species within the genus of interest that are present in each study area, which was never more than three species. For another five species, including *Brugmansia* spp., *Castilleja* spp., *Chuquiraga jussieui*, *Vaccinium floribundum*, and *Berberis* spp., nectar data were not available, although the rest of the floral traits were included in the analysis. Moreover, we lacked floral trait data for *Stenomesson auriantiacum*.

### 2.5 Data Analysis

We first performed a community ordination analysis to evaluate differences in community composition across sites and sampling periods. This analysis was performed using a non‐metric multidimensional scaling based on Bray‐Curtis dissimilarities. We used the number of flowers of each species found in each site and each sampling period. In addition, we conducted a PERMANOVA to statistically test for differences in community composition among sites and sampling periods.

We assessed the associations between node degree—in and out—of species and their abundances and floral traits using linear mixed models. The node degree of the different species was used as the response variable, while the floral traits (stigma and stamen exertion, corolla opening, floral tube length, nectar concentration, and nectar production) and floral abundance were used as the fixed factors; we used network identity as a random factor to consider the potential correlation of species that are part of the same network. We conducted univariate variable models using the lmer function from the lme4 package in R (Bates et al. 2015). To assess predictor significance, we used the lmerTest package (Kuznetsova et al. 2017), which provides t‐statistics and *p*‐values for fixed effects based on Satterthwaite's approximation of denominator degrees of freedom. In these models, we excluded *Passiflora cumbalensis*; its flower is up to 20 cm in length, which is 2 times larger than the second largest species; therefore, including this species would have influenced the normality of the distribution of the fixed factors in the models. We also examined pairwise correlations among floral traits. These correlations revealed functional trade‐offs and associations, such as a negative relationship between tube length and nectar production, and a decoupling between stigma and stamen exertion (Figure S1).

Finally, in order to assess the potential implications of floral roles on the fitness of the different species, we calculated the conspecific‐to‐heterospecific pollen grains ratio (CP/HP ratio). This was done by computing the mean values of conspecific and heterospecific pollen grains for each sample of each species. An increase in the CP/HP ratio suggests a higher proportion of conspecific versus heterospecific pollen grains, potentially indicating positive fitness outcomes. Conversely, a decrease in the ratio implies a higher proportion of heterospecific versus conspecific pollen grains, indicating negative consequences for the fitness of the pollen‐sharing role of the species. We constructed linear mixed models using node degree as the response variable and CP/HP ratio as a fixed factor; locality was a random factor grouping species that are part of the same network. The use of CP/HP ratio should only be considered a broad indicator of the fitness consequences of sharing pollen because there are other measures that are direct indicators of fitness effects (e.g., pollen tube growth, seed set). However, this measure can be a useful indicator across species with the premise that it will always be beneficial for a species to receive more conspecific pollen than heterospecific pollen (Lanuza et al. 2021), considering that even small amounts of heterospecific pollen can reduce reproductive success (Thomson et al. 1982). We did not model node degree out with CP/HP ratio because the fitness consequences of donor roles should be mostly related to male fitness (pollen waste), a fitness measure out of the scope of our analysis.

## 3 Results

Overall, we collected 1454 stigmas of 31 flower species. On these stigmas, we identified 5341 hetero‐specific pollen grains from 14 families and 38 species; 75.98% of the hetero‐specific pollen grains were identified to the species level, 15% to the genus level, and 7.62% to family level; 1.6% of the heterospecific pollen were not identified and thus not used in later analysis. Of all the pollen grains observed, 97.2% were con‐specific (CP) and 2.8% were hetero‐specific (HP). Additionally, on average, each species received 191 conspecific pollen grains and only 3.02 heterospecific pollen grains (Table S1).

**FIGURE 2:**
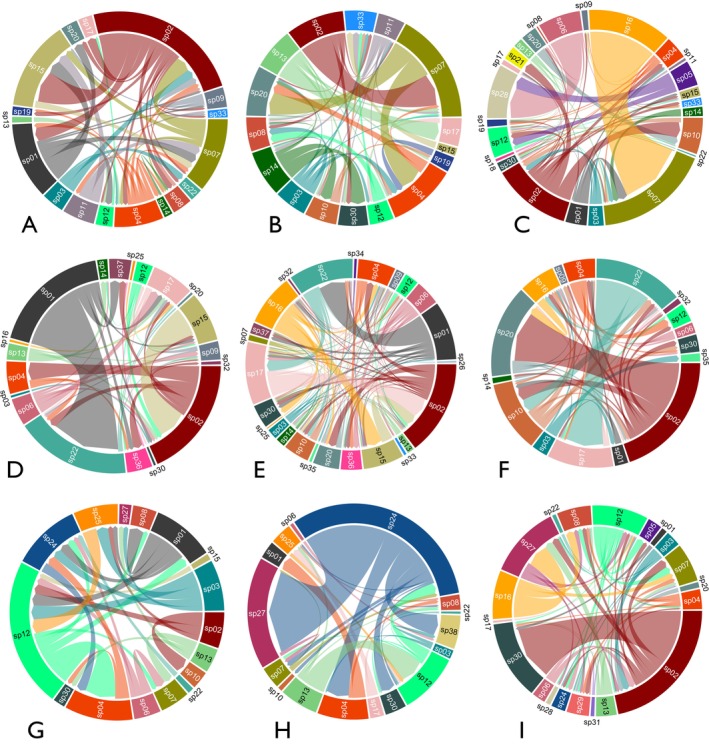
Chord diagrams of indirect interaction networks mediated by hummingbirds in three localities of the southern Andes of Ecuador: La Tranca (A–C), Aguarongo (D–F), and El Gullán (G–I). Each locality was sampled during three periods: August 2022 (A, D and G), November 2022 (B, E and H), and January 2023 (C, F and I). The size of the nodes and arrows denotes the prevalence of interspecific pollen transfer as determined from the median number of heterospecific pollen grains encountered on the stigmas of each plant species. The direction of the tips of the arrows indicates the direction of the interaction. Species names and codes are also presented in Table S2.

La Tranca network was built with 25 nodes connected by 116 arrows, with 24.14% of the arrows being reciprocal. The Aguarongo network had 25 nodes connected to 114 arrows, showing 28% of reciprocal arrows. The El Gullán network had 20 nodes connected to 90 arrows (Figure 2), of which 20% were reciprocal.

To further explore differences in community structure and their potential effects on floral roles, we performed a NMDS analysis which revealed clear separation among communities across sites and sampling periods. This visual pattern was confirmed by PERMANOVA results, which indicated significant variation in species composition (*R* ^2^ = 0.432, *F* = 2.29, *p* = 0.003). These results suggest that variation in species presence and abundance across space and time likely influenced the floral roles in different networks (Table S6 and Figure S2).

### 3.1 Floral Roles

**FIGURE 3:**
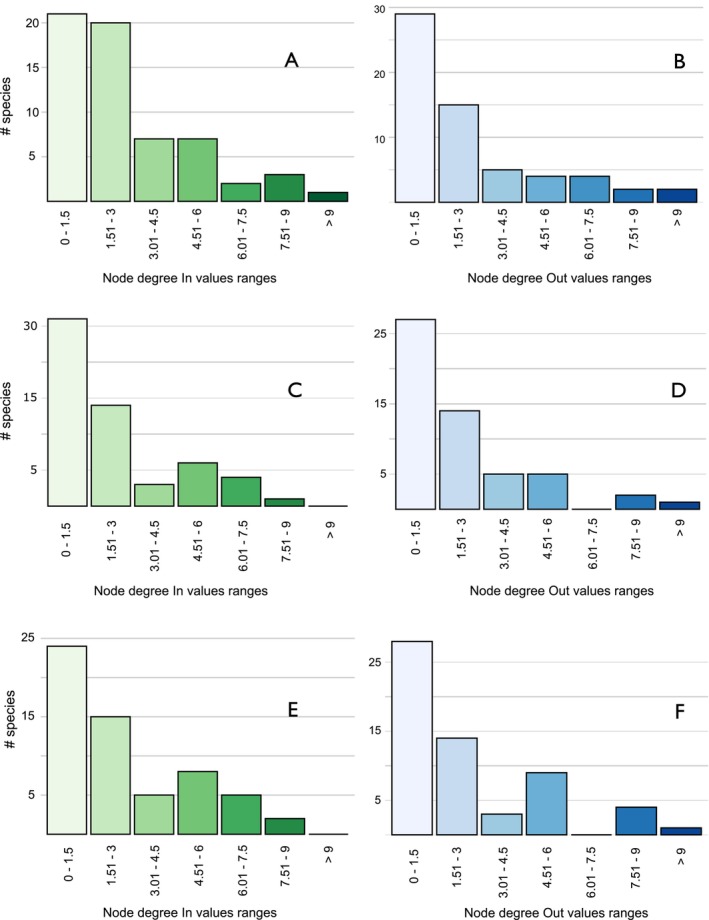
Frequency of species of flowering plants that share pollen grouped by node degree in (A, C, E) and node degree out (B, D, F) in localities of La Tranca (A, B), El Gullán (C, D), and Aguarongo (E, F). The data come from indirect interaction networks mediated by hummingbirds in shrubby habitats of southern Ecuador (Tables S7 and S8).

We found that only a few species exhibited multiple connections to many other species, functioning either as receivers or donors. Meanwhile, the majority of species displayed low node degree in and out values, indicating limited connections to few other species (Figure 3).

**FIGURE 4:**
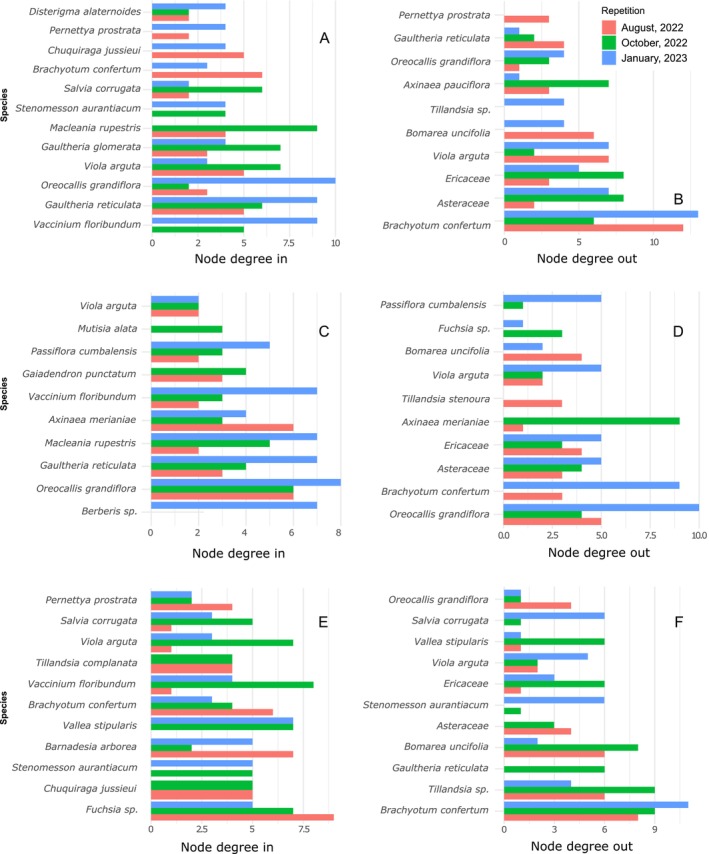
Values of node degree in and node degree out of plant species in three localities La Tranca (A, B), El Gullán (C, D), and Aguarongo (E, F) studied in three different sampling periods (different colors of the bars). The data come from indirect interaction networks mediated by hummingbirds in three shrubby habitats of southern Ecuador (Tables S7 and S8). For each plot, the six species with the highest node degree in and out values were selected and displayed.

There was a large variation in the roles of species among sites, with most species being either an important donor or receiver only in one site, while other species functioned exclusively as either donors or receivers, depending on the location and sampling period (Figure 4). For example, *Macleania rupestris* and *Gaultheria reticulata* acted exclusively as receivers in both La Tranca and El Gullan (Figure 4A,C), and *Bomarea uncifolia* acted as a donor in La Tranca and Aguarongo (Figure 4B,F). In contrast, a few species acted simultaneously as both donors and receivers within the same locality, such as *Viola arguta* in La Tranca (Figure 4A,B), *Oreocallis grandiflora* in El Gullan (Figure 4C,D), and *Brachyotum confertum* in Aguarongo (Figure 4E and 4F).

The dynamic of species roles was supported by the weak correlation found between node degree in and out (*S* = 1,030,615, *p* = 0.021, rho = −0.173), suggesting that acting as a donor did not necessarily imply a reciprocal role as a receiver.

### 3.2 Floral Traits and Floral Abundance

**FIGURE 5:**
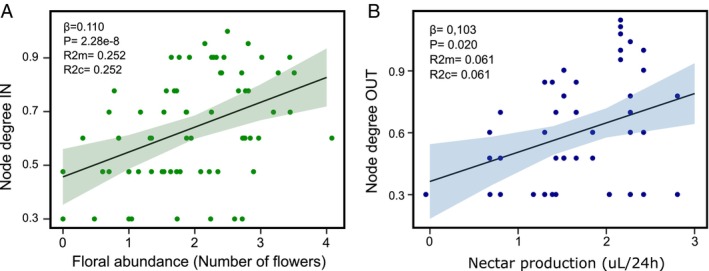
Significant associations between floral abundance and node degree IN (A) and nectar production and node degree OUT (B). Floral abundance, nectar production, and node degree ‐in and out‐ are in log scale. For panel (B) nectar production was added +1 in order to improve the visualization of the plot and evade negative log values. Floral traits with no significant associations are in Figures S3 and S4.

Despite including all floral traits, only abundance and nectar production showed significant associations with floral roles (Tables S3 and S4). Flower abundance was an important predictor of node degree in, indicating that the most abundant species were receptors (Figure 5A). Conversely, species producing high amounts of nectar tended to have higher node degree out, reflecting a stronger role as donor species (Figure 5B). These were the only variables that were significant predictors of node degree in and node degree out (Tables S3 and S4).

### 3.3 Potential Fitness Consequences

**FIGURE 6:**
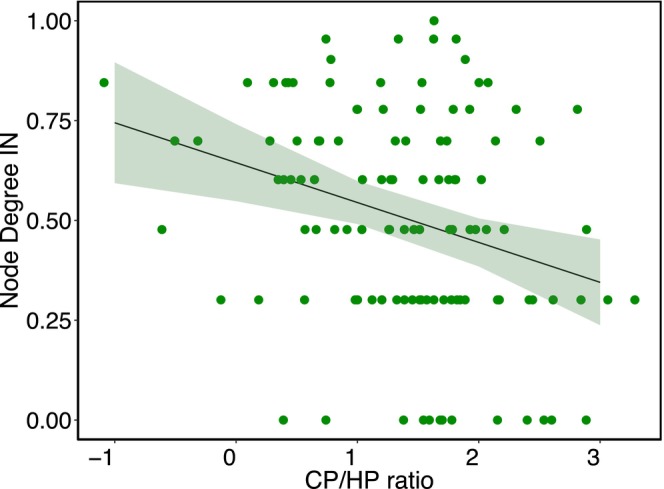
Relationships between the amount of con‐specific (CP) per heterospecific (HP) pollen grains (CP/HP ratio) found in stigmas and node degree of plant species that are part of indirect interaction networks in the southern Andes of Ecuador. The fitted line and confidence intervals (shaded area) represent fitted values from a linear mixed‐effects regression model. Node degree IN and CP/HP ratio are in LOG scale.

We found a significant association between the node degree of the species and CP/HP ratio (df = 114, *t* = −3.31, *p* = 0.001) (Figure 6 and Table S5). This indicates that being a receiver species could lead to negative fitness consequences.

## 4 Discussion

Identifying the roles of plant species that share pollen, and how these roles are influenced by floral traits in indirect plant–plant networks can provide insight into interaction dynamics that potentially impact plant reproductive success and community structure. We found that the role of species in indirect plant–plant networks is highly dynamic in space and time in the tropical Andes. Moreover, the pollen sharing roles were explained by the abundance of flowers and nectar production. Additionally, we found that species that act as pollen receivers could potentially have negative fitness consequences.

In our study, we found an average of 3.13 heterospecific pollen grains per flower, a relatively low number compared to the global patterns of heterospecific pollen on stigmas reported by Arceo‐Gómez et al. (2019). However, the spatial coverage of that global study was low, particularly in tropical regions, where more data are needed for a better understanding of patterns of heterospecific pollen loads. Nonetheless, the relatively low number of heterospecific pollen arriving at the stigmas in our study site suggests high levels of pollinator niche partitioning among plant species. This pattern could be related to the high specialization of tropical Andean hummingbirds in their floral visits (Dalsgaard et al. 2021; Sonne et al. 2016; Weinstein and Graham 2017), which likely reduces pollen mixing (Lopes et al. 2022). Additionally, floral morphological traits, such as the position of anthers, may facilitate pollen placement on distinct body parts of pollinators, further limiting heterospecific pollen transfer (Arteaga‐Chávez et al. 2025; Stewart and Dudash 2016). More studies are needed to identify the mechanisms that influence heterospecific pollen transfer (Arceo‐Gómez et al. 2019; Lopes et al. 2022), but our findings suggest reduced pollen mixing among co‐flowering plants, which may minimize competition for pollinators and facilitate coexistence in the diverse plant communities of the tropical Andes.

Few species acted as receivers or donors of pollen, and these species were connected to multiple species that had weak roles. This pattern fits our predictions and has been observed in other plant–plant interaction networks (Fang and Huang 2013; Tur et al. 2016). An asymmetric assembly of species in networks, where a few species are connected to many species and most species are connected to a few species (Bascompte 2007), is a common pattern in plant–animal mutualistic networks (Jordano et al. 2009), and likely also applies to indirect interaction networks. This asymmetric assembly increases redundancy in ecological networks (Bascompte and Scheffer 2023). If one donor or receiver were to disappear, there are other species to fulfill similar functions, enhancing the stability of the network to perturbations (Thébault and Fontaine 2010). The asymmetric structure in species interactions indicates a hierarchical structure and cohesive organization of networks around a central role of a few species, a pattern that promotes network stability (Bascompte and Jordano 2006).

Flowers exhibited dynamic roles as donors or receivers across sampling locations and sampling periods, indicating that floral roles are context dependent and shaped by spatio‐temporal variation in species presence and abundance. Consistent with this pattern, we found a weak correlation between node degree in and out. This weak association suggests that species can simultaneously act as both donors and receivers, and that the extent to which they donate or receive pollen depends on the local floral community, which changes across space and time. Such flexibility, whereby most species alternate or combine both roles while only a few are restricted to a single role, likely reflects the spatial and temporal variation in plant communities. Spatial variation in floral roles, observed even among proximate areas, can be attributed to a high beta diversity of plants in the tropical Andes (Egawa et al. 2020; Ogishima et al. 2022; Van Der Niet et al. 2014), as it was also the case in our study sites, where the composition of flowers varied among sites (Figure S2 and Table S6). Moreover, floral phenology, predominantly dictated by rainfall‐related events (Günter et al. 2008), shapes the temporal abundance of flowers available for pollinators, consequently affecting the patterns of pollen sharing among plants (Gallagher and Campbell 2020). It is important to mention that this study focuses on plant species known to interact mainly with hummingbirds, and the local roles found may change if a broader range of plant species is included. Nonetheless, despite this limitation, the observed fluctuations in floral roles highlight the dynamic nature of ecological interactions in the tropical Andes.

Different floral traits influenced the donor and receiver roles of species in these high‐Andean plant communities. Receiver roles were primarily associated with floral abundance, a result that contrasts with our expectation that abundant species would primarily act as donors due to their numerical dominance. However, abundant flowers can attract a wide diversity of pollinators (Crespo et al. 2022; Fründ et al. 2010), which may deposit a high diversity of pollen in the stigmas of these species (Peuker et al. 2020). Moreover, donor species were associated with high nectar production. It is likely that pollinators spend longer periods foraging at these nectar‐rich flowers, increasing the amount of pollen they pick up and subsequently transfer to other plants in the community. Hummingbirds have high energetic demands compared to other pollinators and prefer flowers that provide high nectar volumes (Stiles and Freeman 1993). Accordingly, these plant species with high nectar production, particularly attractive to hummingbirds, are exporting their pollen to other hummingbird‐visited plants in the community. Our results indicate that while floral abundance may promote generalized pollinator visitation and increase pollen reception, nectar production appears to enhance targeted pollen export through hummingbird visitation. These trait‐based differences help explain asymmetries in pollen flow and highlight the importance of considering both the quantity and quality of floral rewards in determining floral roles.

There are other floral traits that have been found to be important predictors of floral roles in other studies. For instance, flowers with exserted stigmas tend to accumulate a richness of heterospecific pollen (Ashman and Wei 2024; Fang and Huang 2013; Lanuza et al. 2021). Moreover, floral traits such as corolla opening and floral tube length can filter the species of pollinators that visit a flower and influence the pollen sharing role of species (Fang and Huang 2013; Zhao et al. 2025). However, our study focused on a specific subset of flowers visited by hummingbirds, which may explain the lack of a clear relationship between some floral traits and floral roles. Nonetheless, the traits explaining reception and donor roles can be highly variable within communities (Arceo‐Gómez et al. 2019), with studies even finding weak predictive power of floral traits determining floral roles (Peuker et al. 2020).

We found a significant negative relationship between node degree in and the CP/HP ratio, indicating that receiver species tend to accumulate heterospecific pollen loads. Heterospecific pollen can interfere with pollen tube growth, clog stigmatic surfaces, or outcompete conspecific grains, ultimately reducing fertilization success and fitness (Morales and Traveset 2008; Streher et al. 2020). Receiving heterospecific pollen represents a “service fee” for sharing pollinators with other species (Tur et al. 2016). However, this cost may be offset if pollinator sharing increases the likelihood of receiving enough conspecific pollen to achieve ovule fertilization (Lopes et al. 2022; Tur et al. 2016). Thus, the balance between heterospecific interference and conspecific pollen delivery should determine the fitness consequences of the reception role. Moreover, our measure of fitness costs does not capture all aspects of reproductive success—such as pollen germination, pollen tube growth, or seed set—which are essential for evaluating fitness outcomes of pollen sharing (Németh and Smith‐Huerta 2003). Future studies incorporating these direct fitness measures could help to better understand the implications of floral roles in pollen‐sharing networks.

## 5 Conclusions

Understanding the role of pollinators mediating indirect interactions among co‐flowering plants is crucial for gaining insights into community structure. Our research from the tropical Andes uncovered an asymmetric assembly pattern within hummingbird‐mediated indirect interactions, revealing a cohesive organization around a small set of interactions. Moreover, floral roles in plant–plant interactions seem to be highly dynamic in space and time, where the spatial and temporal abundance of the flowers can play an important role in the patterns of pollen sharing among flowering plants. Importantly, floral roles could impact plant reproductive success, particularly for receiver species, which can likely influence the reproductive strategy of plants. Identifying floral roles enables the identification of species and structures of networks that can contribute to the maintenance and diversity of flowering plants in species‐rich systems.

## Author Contributions

**Ann Frías‐Romero:** conceptualization (equal), data curation (equal), formal analysis (equal), investigation (equal), methodology (equal), writing – original draft (equal). **Boris A. Tinoco:** conceptualization (equal), funding acquisition (lead), investigation (equal), project administration (lead), supervision (lead), writing – review and editing (equal). **Bryan G. Rojas:** methodology (equal), writing – review and editing (equal). **Ariana Vélez:** methodology (equal), writing – review and editing (equal). **Samara Zeas‐Bermeo:** methodology (equal), writing – review and editing (equal). **Catherine H. Graham:** funding acquisition (lead), investigation (equal), supervision (lead), writing – review and editing (equal).

## Conflicts of Interest

The authors declare no conflicts of interest.

## Supporting information


**Data S1:** Supporting Information.

## Data Availability

Data are available from the Dryad Digital Repository: http://doi.org/10.5061/dryad.z08kprrsq.
