# Supplementary material for: Floral Roles in Hummingbirds‐Mediated Indirect Plant Interactions in Tropical Andean Communities
Source: Ecol Evol. 2025 Sep 30;15(10):e72200. doi: 10.1002/ece3.72200 (PMC12483984; doi:10.1002/ece3.72200)
Supplement: Supplementary file 1 — Data S1: Supporting Information. [file ECE3-15-e72200-s001.zip › Supporting Information.docx]

**Supporting information**

*Table S1.* ***General information about the number of samples and pollen grains found on the network.***

|  | TOTAL | Locality | | |
| --- | --- | --- | --- | --- |
|  |  | Aguarongo | El Gullán | La Tranca |
| Number of (species) | 31 | 22 | 18 | 20 |
| Number of samples | 1 454 | 441 | 433 | 580 |
| Number of pollen grain | 191 058 | 70 570 | 68 233 | 52 255 |
| Con-specific pollen grain | 185 717 | 69 002 | 66 700 | 50 015 |
| Average number of Con-specific pollen grains per species | 237 | 159 | 340 | 96.2 |
| Hetero-specific pollen grain | 5341 | 1 568 | 1 533 | 2 240 |
| Average number of Hetero-specific pollen grains per species | 3.11 | 1.83 | 2.15 | 4.95 |

*Table S2.* **List of node names with their corresponding plotcode for identification of the species present in the networks of figure 2.** Species in bold are the species that lack data.

| **Plotcode** | **Node names** | **Plotcode** | **Node names** |
| --- | --- | --- | --- |
| sp01 | *Bomarea uncifolia* | sp20 | *Salvia corrugata* |
| sp02 | *Brachyotum confertum* | sp21 | *Disterigma empetrifolium* |
| sp03 | *Ericaceae* | sp22 | *Barnadesia arborea* |
| sp04 | *Viola arguta* | sp23 | *Lamiaceae* |
| **sp05** | ***Castilleja sp.*** | sp24 | *Axinaeae merianiae* |
| **sp06** | ***Tillandsia sp.*** | sp25 | *Gaiadendron punctatum* |
| sp07 | *Gaultheria reticulata* | sp26 | *Tristerix longebracteatus* |
| sp08 | *Macleania rupestris* | sp27 | *Passiflora cumbalensis* |
| sp09 | *Pernettya prostrata* | sp28 | *Bejaria resinosa* |
| **sp10** | ***Stenomesson aurantiacum*** | **sp29** | ***Berberis sp.*** |
| sp11 | *Axinaeae pauciflora* | **sp30** | ***Vaccinium floribundum*** |
| sp12 | *Oreocallis grandiflora* | **sp31** | ***Brugmansia sp.*** |
| sp13 | *Asteraceae* | sp32 | *Gaultheria erecta* |
| **sp14** | ***Rubus sp.*** | sp33 | *Gaultheria glomerata* |
| sp15 | *Chuquiragua jussieui* | **sp34** | ***Nasa sp.*** |
| sp16 | *Vallea stipularis* | sp35 | *Tillandsia buserii* |
| **sp17** | ***Fuchsia sp.*** | sp36 | *Tillandsia complanata* |
| **sp18** | ***Centropogon sp.*** | sp37 | *Tillandsia stenoura* |
| sp19 | *Disterigma alaternoides* | sp38 | *Mutisia alata* |

*Table S3.* ***Linear model estimates for floral traits and node degree in values.***

| **Node degree in** | | | | | |  |  |
| --- | --- | --- | --- | --- | --- | --- | --- |
|  | **Estimate** | **Std. Error** | **Degree freedom** | **t-value** | **P** | **R2m** | **R2c** |
| **Floral Abundance** | **0.110** | **0.018** | **109** | **6.03** | **<0.001** | **0.252** | **0.252** |
| **Opening corolla** | -0.05157 | 0.05108 | 102 | -1.01 | 0.315 | 0.01 | 0.01 |
| **Floral tube length** | 0.0081 | 0.051 | 102 | 0.16 | 0.874 | 0.0002 | 0.0002 |
| **Stamen exertion** | 0.02771 | 0.05489 | 34.18 | 0.505 | 0.617 | 0.007 | 0.015 |
| **Nectar concentration** | -0.04651 | 0.16 | 90 | -0.290 | 0.772 | 0.0009 | 0.0009 |
| **Stigma exertion** | 0.01837 | 0.03020 | 59 | 0.608 | 0.545 | 0.006 | 0.006 |
| **Nectar production** | 0.02995 | 0.03092 | 99 | 0.969 | 0.335 | 0.010 | 0.010 |

***Table S4.*** *Linear model estimates for floral traits and node degree out values.*

| **Node degree out** | | | | | |  |  |
| --- | --- | --- | --- | --- | --- | --- | --- |
|  | **Estimate** | **Std. Error** | **Degree freedom** | **t-value** | **P** | **R2m** | **R2c** |
| **Floral Abundance** | -0.03227 | 0.03422 | 75 | - 0.943 | 0.349 | 0.012 | 0.012 |
| **Opening corolla** | 0.08435 | 0.06738 | 92 | 1.252 | 0.214 | 0.017 | 0.017 |
| **Floral tube length** | 0.087 | 0.064 | 92 | 1.362 | 0.176 | 0.019 | 0.019 |
| **Stamen exertion** | 0.007 | 0.068 | 40 | 0.11 | 0.913 | 0.0003 | 0.0003 |
| **Nectar concentration** | -0.4012 | 0.2110 | 87 | -1.901 | 0.061 | 0.040 | 0.040 |
| **Stigma exertion** | -0.01830 | 0.04222 | 64 | -0.433 | 0.666 | 0.003 | 0.003 |
| **Nectar production** | **0.10332** | **0.04363** | **87** | **2.368** | **0.020** | **0.061** | **0.061** |

*Table S5.* ***Linear model estimates for CP/HP ratio and node degree in.***

| Node degree IN | | | | | | | |
| --- | --- | --- | --- | --- | --- | --- | --- |
|  | Estimate | Std. Error | Degree freedom | t-value | P | R2m | R2c |
| CP/HP ratio | -0.09992 | 0.03019 | 114 | -3.31 | 0.00125 | 0.088 | 0.088 |

*Table S6.* ***Results of the Permanova analysis assessing differences in community composition.*** *The model indicates significant differences in community composition among groups.*

|  | Df | sumofSqa | R2 | F | Pr |
| --- | --- | --- | --- | --- | --- |
| Model | 2 | 0.92426 | 0.43247 | 2.2861 | 0.003* |
| Residual | 6 | 1.21291 | 0.56753 |  |  |
| Total | 8 | 2.13717 | 1 |  |  |

*Table S7.* **Node degree in for species found at each site and sampling period.**

| ***Site/Repetition***  ***Species*** | **Aguarongo** | | | **El Gullán** | | | **La Tranca** | | |
| --- | --- | --- | --- | --- | --- | --- | --- | --- | --- |
|  | 1 | **2** | **3** | **1** | **2** | **3** | **1** | **2** | **3** |
| ***Asteraceae*** | 0 | 0 |  | 0 | 0 | 0 | 0 | 0 | 0 |
| ***Axinaea merianiae*** |  |  |  | 6 | 3 | 4 |  |  |  |
| ***Axinaea pauciflora*** |  |  |  |  |  |  | 1 | 2 | 0 |
| ***Barnadesia arborea*** | 7 | 2 | 5 | 0 | 0 | 0 | 2 |  | 0 |
| ***Bejaria resinosa*** |  |  |  |  |  | 2 |  |  | 2 |
| ***Berberis sp.*** |  |  |  |  |  | 7 |  |  |  |
| ***Bomarea uncifolia*** | 0 | 0 | 1 | 1 | 1 | 0 | 2 |  | 2 |
| ***Brachyotum confertum*** | 6 | 4 | 3 | 0 |  | 6 | 6 | 0 | 3 |
| ***Brugmansia sp.*** |  |  |  |  |  | 0 |  |  |  |
| ***Castilleja sp.*** |  |  |  |  |  | 2 |  |  | 0 |
| ***Centropogon sp.*** |  |  |  |  |  |  |  |  | 0 |
| ***Chuquiraga jussieui*** | 5 | 5 |  | 0 |  |  | 5 | 0 | 4 |
| ***Disterigma alaternoides*** |  |  |  |  |  |  | 2 | 2 | 4 |
| ***Disterigma empetrifolium*** |  |  |  |  |  |  |  |  | 2 |
| ***Ericaceae*** | 0 | 0 | 0 | 0 | 0 | 0 | 0 | 0 | 0 |
| ***Fuchsia sp.*** | 9 | 7 | 5 |  | 0 | 0 | 2 | 2 | 3 |
| ***Gaiadendron punctatum*** | 0 |  |  | 3 | 4 |  |  |  |  |
| ***Gaultheria erecta*** | 1 |  | 2 |  |  |  |  |  |  |
| ***Gaultheria glomerata*** |  | 2 |  |  |  |  | 3 | 7 | 4 |
| ***Gaultheria reticulata*** |  | 0 |  | 3 | 4 | 7 | 5 | 6 | 9 |
| ***Macleania rupestris*** |  |  |  | 2 | 5 | 7 | 4 | 9 | 0 |
| ***Mutisia alata*** |  |  |  |  | 3 |  |  |  |  |
| ***Nasa sp.*** |  | 0 |  |  |  |  |  |  |  |
| ***Oreocallis grandiflora*** | 1 | 3 | 2 | 6 | 6 | 8 | 3 | 2 | 10 |
| ***Passiflora cumbalensis*** |  |  |  | 2 | 3 | 5 |  |  |  |
| ***Pernettya prostrata*** | 4 | 2 | 2 |  |  |  | 2 |  | 4 |
| ***Rubus sp.*** | 3 | 2 | 2 |  |  |  | 1 | 3 | 0 |
| ***Salvia corrugata*** | 1 | 5 | 3 |  |  | 0 | 2 | 6 | 2 |
| ***Sin identificar*** | 0 | 0 |  |  | 0 | 0 | 0 | 0 | 0 |
| ***Stenomesson aurantiacum*** |  | 5 | 5 | 1 | 1 |  |  | 4 | 4 |
| ***Tillandsia buseri*** |  |  | 1 |  |  |  |  |  |  |
| ***Tillandsia complanata*** | 4 | 4 |  |  |  |  |  |  |  |
| ***Tillandsia sp.*** | 0 | 0 | 0 |  | 0 | 0 |  |  | 0 |
| ***Tillandsia stenoura*** | 3 | 2 |  | 0 |  |  |  |  |  |
| ***Tristerix longebracteatus*** |  | 0 |  |  |  |  |  |  |  |
| ***Vaccinium floribundum*** | 1 | 8 | 4 | 2 | 3 | 7 |  | 5 | 9 |
| ***Vallea stipularis*** | 0 | 7 | 7 |  |  | 0 |  |  | 0 |
| ***Viola arguta*** | 1 | 7 | 3 | 2 | 2 | 2 | 5 | 7 | 3 |

Table S8*.* ***Node degree out for species found at each site and sampling period.***

| ***Site/Repetition***    ***Species*** | **Aguarongo** | | | **El Gullán** | | | **La Tranca** | | |
| --- | --- | --- | --- | --- | --- | --- | --- | --- | --- |
|  | 1 | **2** | **3** | **1** | **2** | **3** | **1** | **2** | **3** |
| *Asteraceae* | 4 | 3 |  | 3 | 4 | 5 | 2 | 8 | 7 |
| *Axinaea merianiae* |  |  |  | 1 | 9 | 0 |  |  |  |
| *Axinaea pauciflora* |  |  |  |  |  |  | 3 | 7 | 1 |
| *Barnadesia arborea* | 3 | 2 | 3 | 1 | 1 | 2 | 0 |  | 1 |
| *Bejaria resinosa* |  |  |  |  |  | 0 |  |  | 0 |
| *Berberis sp.* |  |  |  |  |  | 1 |  |  |  |
| *Bomarea uncifolia* | 6 | 8 | 2 | 4 | 0 | 2 | 6 |  | 4 |
| *Brachyotum confertum* | 8 | 9 | 11 | 3 |  | 9 | 12 | 6 | 13 |
| *Brugmansia sp.* |  |  |  |  |  | 2 |  |  |  |
| *Castilleja sp.* |  |  |  |  |  | 0 |  |  | 2 |
| *Centropogon sp.* |  |  |  |  |  |  |  |  | 2 |
| *Chuquiraga jussieui* | 1 | 0 |  | 1 |  |  | 1 | 1 | 0 |
| *Disterigma alaternoides* |  |  |  |  |  |  | 0 | 1 | 0 |
| *Disterigma empetrifolium* |  |  |  |  |  |  |  |  | 0 |
| *Ericaceae* | 1 | 6 | 3 | 4 | 3 | 5 | 3 | 8 | 5 |
| *Fuchsia sp.* | 3 | 5 | 3 |  | 3 | 1 | 0 | 2 | 1 |
| *Gaiadendron punctatum* | 1 |  |  | 1 | 1 |  |  |  |  |
| *Gaultheria erecta* | 0 |  | 0 |  |  |  |  |  |  |
| *Gaultheria glomerata* |  | 0 |  |  |  |  | 0 | 0 | 0 |
| *Gaultheria reticulata* |  | 6 |  | 0 | 2 | 0 | 4 | 2 | 1 |
| *Macleania rupestris* |  |  |  | 0 | 1 | 0 | 1 | 1 | 1 |
| *Mutisia alata* |  |  |  |  | 0 |  |  |  |  |
| *Nasa sp.* |  | 2 |  |  |  |  |  |  |  |
| *Oreocallis grandiflora* | 4 | 1 | 1 | 5 | 4 | 10 | 1 | 3 | 4 |
| *Passiflora cumbalensis* |  |  |  | 0 | 1 | 5 |  |  |  |
| *Pernettya prostrata* | 2 | 0 | 0 |  |  |  | 3 |  | 0 |
| *Rubus sp.* | 0 | 2 | 0 |  |  |  | 1 | 4 | 3 |
| *Salvia corrugata* | 0 | 1 | 6 |  |  | 1 | 0 | 1 | 3 |
| *Sin identificar* | 3 | 1 |  |  | 3 | 3 | 1 | 6 | 3 |
| *Stenomesson aurantiacum* |  | 1 | 6 | 0 | 0 |  |  | 3 | 1 |
| *Tillandsia buseri* |  |  | 0 |  |  |  |  |  |  |
| *Tillandsia complanata* | 1 | 0 |  |  |  |  |  |  |  |
| *Tillandsia sp.* | 6 | 9 | 4 |  | 1 | 2 |  |  | 4 |
| *Tillandsia stenoura* | 0 | 0 |  | 3 |  |  |  |  |  |
| *Tristerix longebracteatus* |  | 1 |  |  |  |  |  |  |  |
| *Vaccinium floribundum* | 0 | 0 | 0 | 0 | 0 | 0 |  | 0 | 0 |
| *Vallea stipularis* | 1 | 6 | 1 |  |  | 4 |  |  | 2 |
| *Viola arguta* | 2 | 2 | 5 | 2 | 2 | 5 | 7 | 2 | 7 |


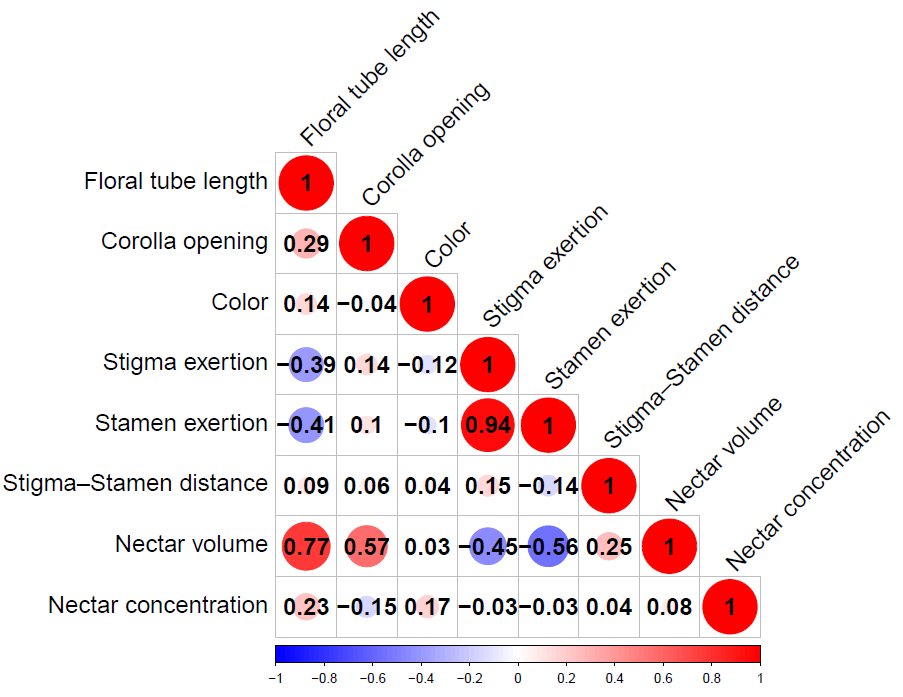


*Figure S1.* ***Heatmap of correlation coefficients among floral traits****. The color and size of the circles indicate the strength and direction of the correlation (red = positive, blue = negative). Strong correlations were found between stigma and stamen exertion, and between floral tube length and nectar volume (R>0.69). Moderate correlation was also observed between stigma and stamen exertion with floral tube length and nectar volume (0.40<R<0.69).*


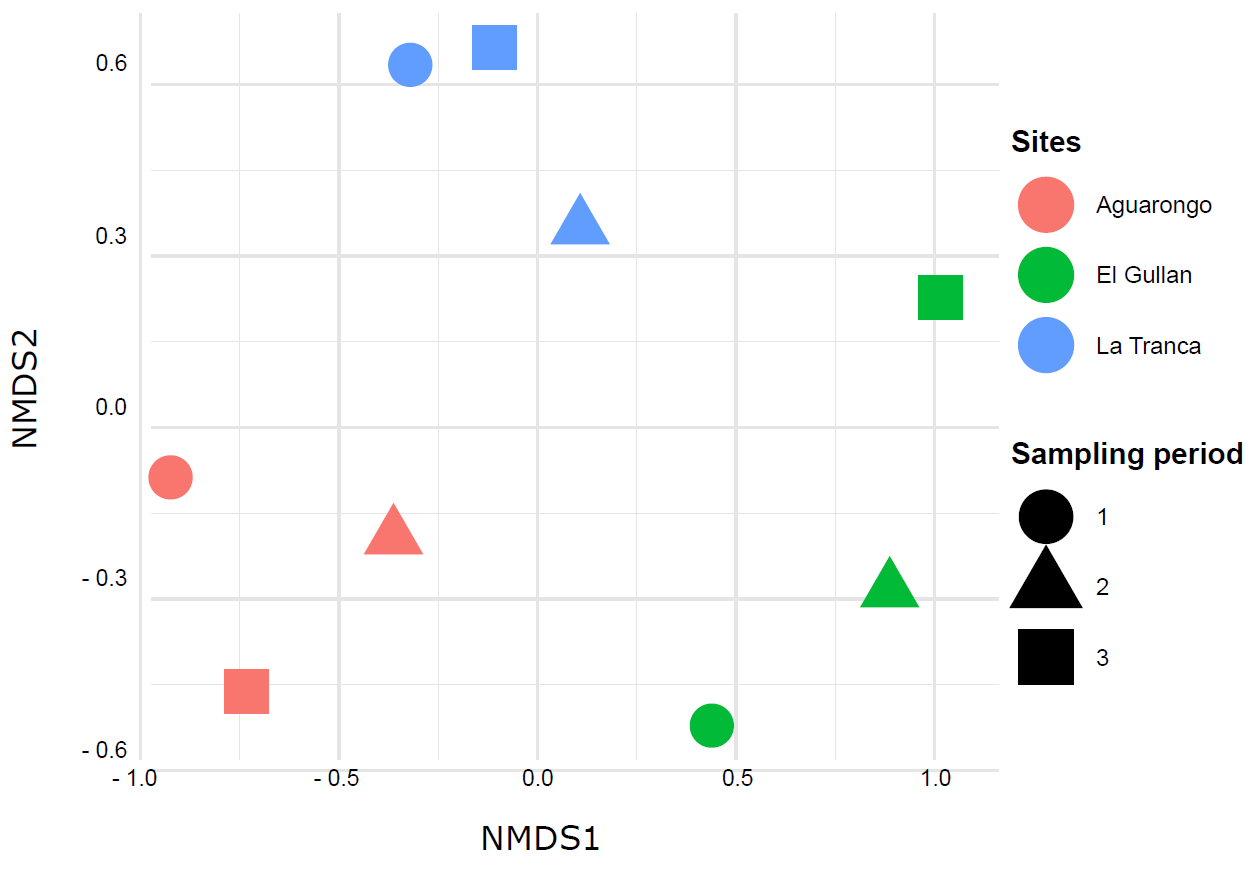


*Figure S2****. Beta-diversity among sampling sites and periods. NMDS ordination based on Bray-Curtis dissimilarities, showing variation in community composition across three sites (Aguarongo, El Gullán and La Tranca) and three sampling periods.*** *Each point represents the composition of a community based on data of the number of flowers produced by each species, with colors indicating site and shapes indicating sampling period.*


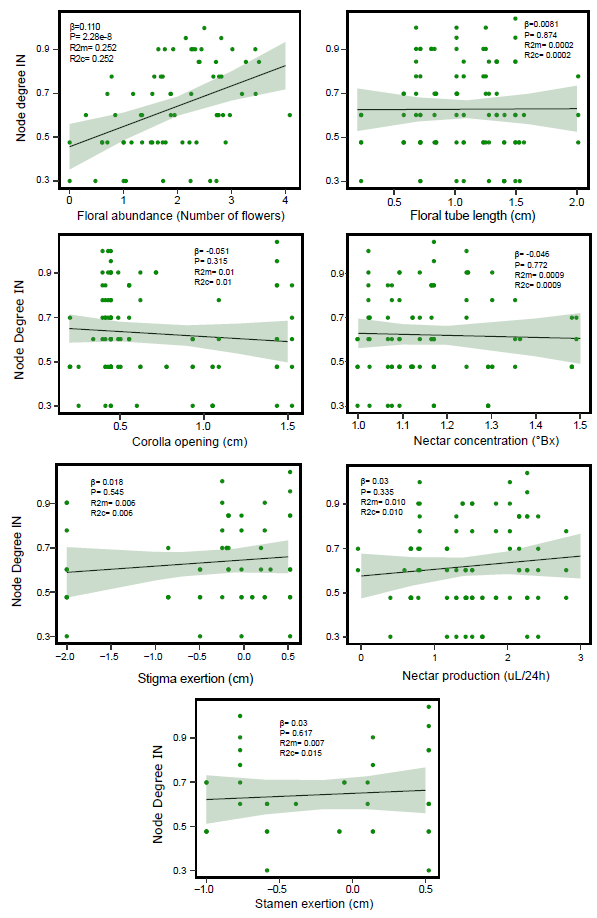


*Figure S3****. Associations between different floral traits and node degree in of flower species that are part of indirect interactions in shrubby habitats of the southern Andes of Ecuador.*** *(A) for floral abundance, (B) for floral tube length, (C) for corolla opening, (D) nectar concentration, (E) for stigma exertion, and (F) for stamen exertion. All predictor variables are in log scale, except for stigma and stamen exertion. Additionally, x axis for floral tube length, corolla opening and nectar production were added +1 to improve the visualization of the plot and evade negative log values. Fitted lines and confidence intervals (shaded area) represent fitted values from linear mixed-effects regression model.*


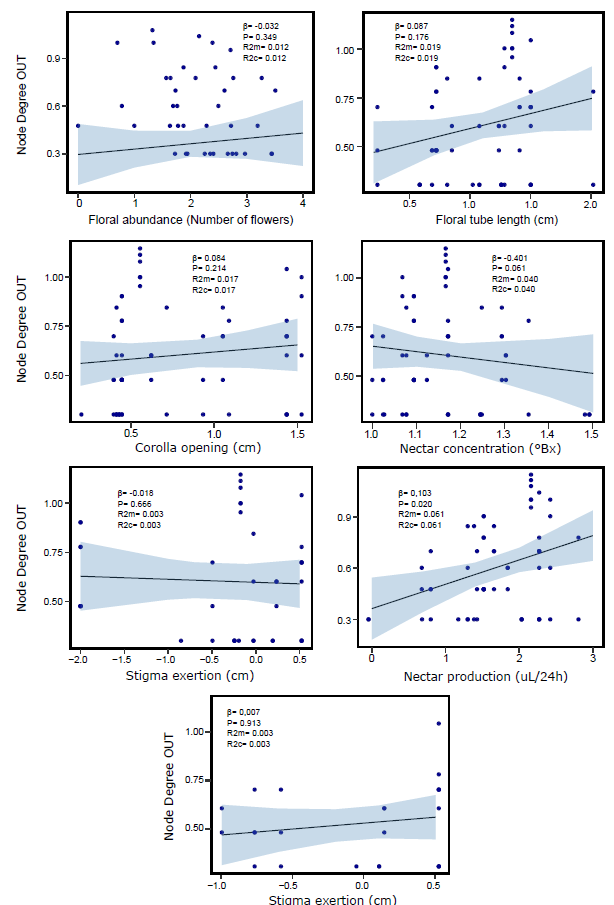


*Figure S4.* ***Associations between different floral traits and node degree out values of flower species that are part of indirect interactions in shrubby habitats of the southern Andes of Ecuador.*** *(A) for floral abundance, (B) for floral tube length, (C) for corolla opening, (D) nectar concentration, (E) for stigma exertion, and (F) for stamen exertion. All predictor variables are in log scale, except for stigma and stamen exertion.* *Additionally, x axis for floral tube length, corolla opening and nectar production were added +1 to improve the visualization of the plot and evade negative log values. Fitted lines and confidence intervals (shaded area) represent fitted values from linear mixed-effects regression model.*
