# Supplementary material for: Floral Roles in Hummingbirds‐Mediated Indirect Plant Interactions in Tropical Andean Communities
Source: Ecol Evol. 2025 Sep 30;15(10):e72200. doi: 10.1002/ece3.72200 (PMC12483984; doi:10.1002/ece3.72200)
Supplement: Supplementary file 1 — Data S1: Supporting Information. [file ECE3-15-e72200-s001.zip › Table S1.pdf]

Appendix table 1. General information about the number of samples and pollen grains found on the network.

	TOTAL	Locality		
		Aguarongo	El Gullán	La Tranca
Number of (species)	31	22	18	20
Number of samples	1 454	441	433	580
Number of pollen grain	191 058	70 570	68 233	52 255
Con-specific pollen grain	185 717	69 002	66 700	50 015
Average number of Con-specific pollen grains per species	237	159	340	96.2
Hetero-specific pollen grain	5341	1 568	1 533	2 240
Average number of Hetero-specific pollen grains per species	3.11	1.83	2.15	4.95
