# Supplementary material for: Floral Roles in Hummingbirds‐Mediated Indirect Plant Interactions in Tropical Andean Communities
Source: Ecol Evol. 2025 Sep 30;15(10):e72200. doi: 10.1002/ece3.72200 (PMC12483984; doi:10.1002/ece3.72200)
Supplement: Supplementary file 1 — Data S1: Supporting Information. [file ECE3-15-e72200-s001.zip › Table S2.pdf]

Appendix table 2. List of node names with their corresponding plotcode for identification of the species present in the networks of figure 2. Species in bold are the species that lack data.

Plotcode	Node names	Plotcode	Node names
sp01	*Bomarea uncifolia*	sp20	*Salvia corrugata*
sp02	*Brachyotum confertum*	sp21	*Disterigma empetrifolium*
sp03	*Ericaceae*	sp22	*Barnadesia arborea*
sp04	*Viola arguta*	sp23	*Lamiaceae*
**sp05**	***Castilleja sp.***	sp24	*Axinaeae merianiae*
**sp06**	***Tillandsia sp.***	sp25	*Gaiadendron punctatum*
sp07	*Gaultheria reticulata*	sp26	*Tristerix longebracteatus*
sp08	*Macleania rupestris*	sp27	*Passiflora cumbalensis*
sp09	*Pernettya prostrata*	sp28	*Bejaria resinosa*
**sp10**	***Stenomesson aurantiacum***	**sp29**	***Berberis sp.***

sp11	*Axinaeae pauciflor a*	**sp30**	***Vaccinium floribundum***
sp12	*Oreocallis grandiflora*	**sp31**	***Brugmansia sp.***
sp13	*Asteraceae*	sp32	*Gaultheria erecta*
**sp14**	***Rubus sp.***	sp33	*Gaultheria glomerata*
sp15	*Chuquiragua jussieui*	**sp34**	***Nasa sp.***
sp16	*Vallea stipularis*	sp35	*Tillandsia buserii*
**sp17**	***Fuchsia sp.***	sp36	*Tillandsia complanata*
**sp18**	***Centropogon sp.***	sp37	*Tillandsia stenoura*
sp19	*Disterigma alaternoides*	sp38	*Mutisia alata*
