# Supplementary material for: Floral Roles in Hummingbirds‐Mediated Indirect Plant Interactions in Tropical Andean Communities
Source: Ecol Evol. 2025 Sep 30;15(10):e72200. doi: 10.1002/ece3.72200 (PMC12483984; doi:10.1002/ece3.72200)
Supplement: Supplementary file 1 — Data S1: Supporting Information. [file ECE3-15-e72200-s001.zip › Table S3.pdf]

Appendix table 3. Linear model estimates for floral traits and node degree in values.

			**Node degree in**				
	**Estimate**	**Std. Error**	**Degree freedom**	**t-value**	**P**	**R2m**	**R2c**
**Floral Abundance**	**0.110**	**0.018**	**109**	**6.03**	**<0.001**	**0.252**	**0.252**
**Opening corolla**	-0.05157	0.05108	102	-1.01	0.315	0.01	0.01
**Floral tube length**	0.0081	0.051	102	0.16	0.874	0.0002	0.0002
**Stamen exertion**	0.02771	0.05489	34.18	0.505	0.617	0.007	0.015
**Nectar concentrati on**	-0.04651	0.16	90	-0.290	0.772	0.0009	0.0009
**Stigma exertion**	0.01837	0.03020	59	0.608	0.545	0.006	0.006
**Nectar production**	0.02995	0.03092	99	0.969	0.335	0.010	0.010
