# Supplementary material for: Floral Roles in Hummingbirds‐Mediated Indirect Plant Interactions in Tropical Andean Communities
Source: Ecol Evol. 2025 Sep 30;15(10):e72200. doi: 10.1002/ece3.72200 (PMC12483984; doi:10.1002/ece3.72200)
Supplement: Supplementary file 1 — Data S1: Supporting Information. [file ECE3-15-e72200-s001.zip › Table S4.pdf]

Appendix table 4. *Linear model estimates for floral traits and node degree out values.*

	Node degree out						
	**Estimate**	**Std. Error**	**Degree freedom**	**t-value**	**P**	**R2m**	**R2c**
**Floral Abundance**	-0.03227	0.03422	75	- 0.943	0.349	0.012	0.012
**Opening corolla**	0.08435	0.06738	92	1.252	0.214	0.017	0.017
**Floral tube length**	0.087	0.064	92	1.362	0.176	0.019	0.019
**Stamen exertion**	0.007	0.068	40	0.11	0.913	0.0003	0.0003
**Nectar concentration**	-0.4012	0.2110	87	-1.901	0.061	0.040	0.040
**Stigma exertion**	-0.01830	0.04222	64	-0.433	0.666	0.003	0.003
**Nectar production**	**0.10332**	**0.04363**	**87**	**2.368**	**0.020**	**0.061**	**0.061**
