# Supplementary material for: Floral Roles in Hummingbirds‐Mediated Indirect Plant Interactions in Tropical Andean Communities
Source: Ecol Evol. 2025 Sep 30;15(10):e72200. doi: 10.1002/ece3.72200 (PMC12483984; doi:10.1002/ece3.72200)
Supplement: Supplementary file 1 — Data S1: Supporting Information. [file ECE3-15-e72200-s001.zip › Table S5.pdf]

Appendix table 5. Linear model estimates for CP/HP ratio and node degree in.

	Node degree IN						
	Estimate	Std. Error	Degree freedom	t-value	P	R2m	R2c
**CP/HP ratio**	-0.09992	0.03019	114	-3.31	0.00125	0.088	0.088
