# Supplementary material for: Floral Roles in Hummingbirds‐Mediated Indirect Plant Interactions in Tropical Andean Communities
Source: Ecol Evol. 2025 Sep 30;15(10):e72200. doi: 10.1002/ece3.72200 (PMC12483984; doi:10.1002/ece3.72200)
Supplement: Supplementary file 1 — Data S1: Supporting Information. [file ECE3-15-e72200-s001.zip › Table S6.pdf]

Appendix table 6. Results of the Permanova analysis assessing differences in community composition. The model indicates significant differences in community composition among groups.

	Df	sumofSqa	R2	F	Pr
**Model**	2	0.92426	0.43247	2.2861	0.003*
**Residual**	6	1.21291	0.56753		
**Total**	8	2.13717	1		
