# Supplementary material for: Floral Roles in Hummingbirds‐Mediated Indirect Plant Interactions in Tropical Andean Communities
Source: Ecol Evol. 2025 Sep 30;15(10):e72200. doi: 10.1002/ece3.72200 (PMC12483984; doi:10.1002/ece3.72200)
Supplement: Supplementary file 1 — Data S1: Supporting Information. [file ECE3-15-e72200-s001.zip › Table S7.pdf]

Appendices table 7. Node degree in for species found at each site and sampling period.

Site/Repetition	Aguarongo			El Gullán			La Tranca		
Species	1	2	3	1	2	3	1	2	3
Asteraceae	0	0		0	0	0	0	0	0
Axinaea merianiae				6	3	4			
Axinaea pauciflora							1	2	0
Barnadesia arborea	7	2	5	0	0	0	2		0
Bejaria resinosa						2			2
Berberis sp.						7			
Bomarea uncifolia	0	0	1	1	1	0	2		2
Brachyotum confertum	6	4	3	0		6	6	0	3
Brugmansia sp.						0			
Castilleja sp.						2			0
Centropogon sp.									0
Chuquiraga jussieui	5	5		0			5	0	4
Disterigma alaternoides							2	2	4
Disterigma empetrifolium									2
Ericaceae	0	0	0	0	0	0	0	0	0

Fuchsia sp.	9	7	5		0	0	2	2	3
Gaiadendron punctatum	0			3	4				
Gaultheria erecta	1		2						
Gaultheria glomerata		2					3	7	4
Gaultheria reticulata		0		3	4	7	5	6	9
Macleania rupestris				2	5	7	4	9	0
Mutisia alata					3				
Nasa sp.		0							
Oreocallis grandiflora	1	3	2	6	6	8	3	2	10
Passiflora cumbalensis				2	3	5			
Pernettya prostrata	4	2	2				2		4
Rubus sp.	3	2	2				1	3	0
Salvia corrugata	1	5	3			0	2	6	2
Sin identificar	0	0			0	0	0	0	0
Stenomesson aurantiacum		5	5	1	1			4	4
Tillandsia buseri			1						
Tillandsia complanata	4	4							
Tillandsia sp.	0	0	0		0	0			0
Tillandsia stenoura	3	2		0					
Tristerix longebracteatus		0							

Vaccinium floribundum	1	8	4	2	3	7		5	9
Vallea stipularis	0	7	7			0			0
Viola arguta	1	7	3	2	2	2	5	7	3
