# Supplementary material for: Floral Roles in Hummingbirds‐Mediated Indirect Plant Interactions in Tropical Andean Communities
Source: Ecol Evol. 2025 Sep 30;15(10):e72200. doi: 10.1002/ece3.72200 (PMC12483984; doi:10.1002/ece3.72200)
Supplement: Supplementary file 1 — Data S1: Supporting Information. [file ECE3-15-e72200-s001.zip › Table S8.pdf]

Appendices table 8. Node degree out for species found at each site and sampling period.

Site/Repetition	**Aguarongo**			**El Gullán**			**La Tranca**		
Species	1	**2**	**3**	**1**	**2**	**3**	**1**	**2**	**3**
Asteraceae	4	3		3	4	5	2	8	7
Axinaea merianiae				1	9	0			
Axinaea pauciflora							3	7	1
Barnadesia arborea	3	2	3	1	1	2	0		1
Bejaria resinosa						0			0
Berberis sp.						1			
Bomarea uncifolia	6	8	2	4	0	2	6		4
Brachyotum confertum	8	9	11	3		9	12	6	13
Brugmansia sp.						2			
Castilleja sp.						0			2
Centropogon sp.									2
Chuquiraga jussieui	1	0		1			1	1	0

Disterigma alaternoides							0	1	0
Disterigma empetrifolium									0
Ericaceae	1	6	3	4	3	5	3	8	5
Fuchsia sp.	3	5	3		3	1	0	2	1
Gaiadendron punctatum	1			1	1				
Gaultheria erecta	0		0						
Gaultheria glomerata		0					0	0	0
Gaultheria reticulata		6		0	2	0	4	2	1
Macleania rupestris				0	1	0	1	1	1
Mutisia alata					0				
Nasa sp.		2							
Oreocallis grandiflora	4	1	1	5	4	10	1	3	4
Passiflora cumbalensis				0	1	5			
Pernettya prostrata	2	0	0				3		0
Rubus sp.	0	2	0				1	4	3
Salvia corrugata	0	1	6			1	0	1	3
Sin identificar	3	1			3	3	1	6	3

Stenomesson aurantiacum		1	6	0	0			3	1
Tillandsia buseri			0						
Tillandsia complanata	1	0							
Tillandsia sp.	6	9	4		1	2			4
Tillandsia stenoura	0	0		3					
Tristerix longebracteatus		1							
Vaccinium floribundum	0	0	0	0	0	0		0	0
Vallea stipularis	1	6	1			4			2
Viola arguta	2	2	5	2	2	5	7	2	7

1
